# Peritoneal mesometrial resection with lymphadenectomy following prior hysterectomy in intermediate/high-risk endometrial cancer: feasibility and safety

**DOI:** 10.1007/s00404-023-07275-3

**Published:** 2024-01-13

**Authors:** Paul Buderath, Mohamed Elgharib, Rainer Kimmig

**Affiliations:** West German Cancer Center, Department for Gynaecology and Obstetrics, University Hospital Essen, University of Duisburg-Essen, Hufelandstr. 55, 45147 Essen, Germany

**Keywords:** Endometrial cancer, Cancer field surgery, Peritoneal mesometrial resection, Sentinel node detection, Targeted compartmental lymphadenectomy

## Abstract

**Objective:**

Peritoneal mesometrial resection (PMMR) plus targeted compartmental lymphadenectomy (TCL) aims at removal of the locoregional cancer field in endometrial cancer (EC). Optimal locoregional control without adjuvant radiotherapy should be achieved concomitantly sparing systematic lymphadenectomy (LNE) for most of the patients. However, intermediate/high-risk EC is often definitely diagnosed postoperatively in simple hysterectomy specimen. Our aim was to evaluate feasibility and safety of a completing PMMR + TCL in patients following prior hysterectomy.

**Methods:**

We evaluated data from 32 patients with intermediate/high-risk EC treated with PMMR + TCL or systematic pelvic and periaortic LNE following prior hysterectomy. Perioperative data on disease characteristics and morbidity were collected and patients were contacted for follow-up to determine the recurrence and survival status.

**Results:**

We report data from 32 patients with a mean follow-up of 31.7 months. The recurrence rate was 12.5% (4/32) without any isolated locoregional recurrences. Only 21.9% of patients received adjuvant radiotherapy. Rates of intra- and postoperative complications were 6.3% and 18.8%, respectively.

**Conclusion:**

Our data suggest that robotic PMMR can be performed following prior hysterectomy when previously unknown risk factors arise, albeit with a moderate increase in morbidity. Moreover, despite a relevant reduction of adjuvant radiotherapy, follow-up data suggest an excellent locoregional control even without adjuvant radiotherapy.

## What does this study add to the clinical work?

**Table:** 

Our study provides first evidence for the feasibility of secondary cancer field surgery following prior simple hysterectomy in high-risk endometrial cancer. It, therefore, contributes to a tailored surgical treatment for these women.	

## Introduction

With around 11.090 newly diagnosed patients per year (forecast for 2020: 10600), endometrial cancer (EC) is the 4th most common cancer in women in developed countries and the most common female genital cancer entity in Germany [1, 2]. In Korea, the age-standardized incidence rate per 100.000 increased from 2.4 in 1999 to 5.7 in 2015 with 2263 new cases diagnosed in 2015 [3]. Despite the overall good prognosis, around 2600 women die of the disease every year in Germany. In most stages, therapy consists of hysterectomy and bilateral salpingo-oophorectomy, followed by adjuvant radio- or chemotherapy according to risk factors [4]. While in low-risk cancers simple hysterectomy followed by observation is sufficient, systematic pelvic and periaortic lymphonodectomy as well as postoperative irradiation is the standard in intermediate/high-risk disease in a lot of countries. In the most recent guidelines, the molecular classification of EC also plays a role in the determination of adjuvant therapy and extent of lymphadenectomy [5]. However, risk factors such as myometrial invasion and definitive tumor grade can only be confirmed in the hysterectomy specimen. Thus, a relevant number of patients presents with intermediate/high-risk EC following simple hysterectomy, raising the question of the optimal treatment strategy for these patients.

We have published the concept and first results of cancer field surgery by peritoneal mesometrial resection (PMMR) and targeted compartmental lymphadenectomy (TCL) in EC earlier [6–9]. To briefly summarize the concept, it is important to understand that EC originates in the embryologically determined Müllerian Compartment. Organ-compartments are derived from common precursor tissues and are topologically organized in defined structures—the so-called morphogenetic fields [10]. During the development of organs and tissues, compartment borders remain and are rigidly controlled within the organism [11]. According to the ontogenetic theory on carcinogenesis, pathological reactivation of normally blocked developmental programs during cancer progression enables the cell to grow outside its own compartment step by step [12]. Whereas tumor growth is, therefore, restricted to a certain compartment for a long time, increasing malignant progression may, thus, facilitate tumor spread across compartment borders. The validity of this theory could already be demonstrated for cervical, vulvar, and rectal cancer [13–15]. The same principles can be applied to the spread of tumor cells to the regional lymph vessels, which belong to the tumor-bearing compartment and originate in the embryonal veins [16].

The aim of ontogenetically defined surgical techniques is, thus, to achieve optimal locoregional control by complete resection of the tumor-bearing compartment (“cancer field”). For cervical and vulvar cancer, the techniques of total mesometrial resection and therapeutic lymphadenectomy (TMMR + tLNE) and of vulvar field resection (VFR) were developed and described on the basis of these concepts [14, 17]. The uni- and multicentric data published so far convincingly support the expectation of excellent locoregional control without adjuvant irradiation [18–20].

In EC, removal of the cancer field consists of complete resection of the Müllerian compartment except of the vagina including the regional draining primary lymph compartments containing the first line nodes. The resulting technique of peritoneal mesometrial resection (PMMR) by minimally invasive, robot-assisted surgery was published in 2013 [9]. First data including therapeutically intended systematic lymphadenectomy indicate excellent local–regional tumor control rates even without postoperative radiotherapy [6, 8].

However, a question of ongoing debate is the role of lymphadenectomy in the surgical treatment of EC due to enhanced perioperative morbidity and development of lymphedema [21]. Diagnostic sentinel lymphadenectomy has become a worldwide standard due to excellent data regarding sensitivity and safety [22, 23]. For the detection of sentinel nodes, indocyanine green (ICG) is regarded as safe and reliable [24–26].

As a consequence, the original concept of PMMR and complete therapeutic pelvic and paraaortic lymphadenectomy has been adapted to a technique of targeted compartmental lymphadenectomy (TCL). This aims in resecting the first line nodes only including the cancer field in continuity from tumor to the nodes identified by the ICG stained draining lymphatic channels and sentinel nodes.

We presented first results of our compartment-based treatment concept via peritoneal mesometrial resection (PMMR) and targeted compartmental lymphadenectomy (TCL) earlier [6]. Given the extremely low morbidity, we offer PMMR + TCL to all EC patients to avoid the above-mentioned conflicts in the case of postoperative upstaging. However, this is not the standard. So, how can patients who underwent a simple hysterectomy for intermediate/high-risk EC benefit from the advantages of cancer field surgery?

Being confronted with this question in our daily practice, we developed the concept of secondary PMMR as completing surgery after prior hysterectomy. ICG is in these cases injected in the vaginal stump and—in case of indications of periaortic TCL—the infundibulopelvic ligament. The aim of the study was to present first data on feasibility and oncologic results of the patients with intermediate/high-risk EC treated by PMMR + TCL/systematic lymphadenectomy after prior simple hysterectomy.

## Materials and methods

To identify patients eligible for analysis, we performed a retrospective systematic search for the ICD-code C.54 (endometrial cancer) for the years 2010 (introduction of the DaVinci surgical system) until 2021 among all surgically treated patients at the department for gynecology and obstetrics in our hospital’s clinical information system. Patients were filtered for their risk classification according to classic histopathological criteria as molecular characteristics were implemented in the national guidelines only recently. Only patients suffering from intermediate/high-risk EC, defined as stage pT1a, G3, and higher pT stages, were included in the analysis. All retrieved patients were manually checked for the procedure performed. Only patients who received peritoneal mesometrial resection (PMMR) including either systematic pelvic ± periaortic lymphadenectomy or targeted compartmental lymphadenectomy (TCL) following prior simple hysterectomy were included in the analysis.

Technically, 1 ml of ICG solution (1.66 mg/ml) were injected into the mesometrium on each side by injection through the right and left sides of the vaginal stump. In the case of periaortic TCL, 1 ml of the same ICG solution was injected into the infundibulopelvic ligament on each side injecting through the abdominal wall under laparoscopic control.

Information on patient characteristics, intra- and perioperative morbidity as well as disease specifications were collected from the electronic patient charts and documented in pseudonymized form. Postoperative complications were classified according to the Clavien–Dindo classification system for postoperative complications [27].

Follow-up information was collected by checking the electronic patient charts for follow-up visits documenting survival and recurrence status. Patients, whose last follow-up was older that three months at the time of analysis were contacted via telephone.

All statistical analyses were performed using SPSS Version 27 (IBM).

## Results

A total of 32 patients matched the inclusion criteria, out of whom 15 had received systematic pelvic ± periaortic lymphadenectomy and 17 were treated by PMMR + TCL. All patients had received prior simple hysterectomy either at our institution or a third-party hospital. All procedures were performed by a single surgeon (RK).

Periaortic nodes were evaluated in all patients who underwent systematic lymphadenectomy and in 4/17 patients (23.5%) in the TCL group.

Mean patient age was 60.8 years (48–80; 7.4), mean BMI 32.4 kg/m^2^ (17–45; 8.4). These characteristics did not differ relevantly between the systematic LNE and TCL groups. Mean length of stay in the whole cohort was 8.8 days (4–30; 5.0). Patients after systematic LNE stayed at the hospital for 11.5 days (5–30; 5.9), while the mean length of stay was 6.4 days (4–11; 2.1) in patients after TCL. Mean skin-to-skin time was 236 min (75–401; 93). As expected, surgery lasted longer when systematic LNE was performed (291 min [150–401; 77.5]) than in the TCL group (187 min [75–393; 78.2]).

The detection rate in the TCL group was 100%. Pelvic sentinel nodes were found at the iliac bifurcation and in the obturator fossa. Periaortic sentinel nodes were located in the interaortocaval region at the mouth of the ovarical veins.

The mean decrease in hemoglobin levels was 2.7 g/dl after systematic LNE compared with 2.1 g/dl after TCL. Patient characteristics are summarized in Table 1.

**Table 1:** Patient characteristics and surgical data

	Total	Syst. LNE	TCL
Age [years]	60.8 (48–80; 7.4) [*n* = 32]	61.5 (53–71; 4.9) [*n* = 15]	60.2 (48–80; 9.1) [*n* = 17]
BMI [kg/m^2^]	32.4 (17–45; 8.4) [*n* = 32]	34.0 (21.5–45; 8.0) [*n* = 15]	30.9 (17–45; 8.7) [*n* = 17]
Follow-up [months]	31.7 (0–92; 28.8) [*n* = 32]	45.8 (0–92; 35.4) [*n* = 15]	19.2 (0–40; 12.6) [*n* = 17]
Length of stay [days]	8.8 (4–30; 5.0) [*n* = 32]	11.5 (5–30; 5.9) [*n* = 15]	6.4 (4–11; 2.1) [*n* = 17]
Skin-to-skin time [min]	236 (75–401; 93) [*n* = 32]	291 (150–401; 77.5) [*n* = 15]	187 (75–393; 78.2) [*n* = 17]
Hb-decrease [g/dl]	− 2.4 (− 0.7–− 7.2; 1.4) [*n* = 31]	− 2.7 (− 1.2–− 5.7; 1.1) [*n* = 15]	− 2.1 (− 0.7–− 7.2; 1.6) [*n* = 16]
Lymph nodes infiltrated	1.3 (0–23; 4.5) [*n* = 32]	2.9 (0–23; 6.3) [*n* = 15]	0 [*n* = 17]
Lymph nodes removed	31.8 (2–99; 26.6) [*n* = 32]	53.7 (28–99; 23.3) [*n* = 15]	12.5 (2–26; 7.0) [*n* = 17]

The overall intraoperative complication rate was 6.3% (2/32). There were no intraoperative complications in the TCL cohort. However, intraoperative complications occurred in 2/15 patients during PMMR with systematic LNE (13.3%). Both complications were vessel lesions which could be managed minimal-invasively. No conversion to open surgery was necessary.

Postoperative complications occurred in 18.8% of patients (6/32). Four of these were classified as Clavien–Dindo stage 3 and higher, indicating the need for operative intervention. Postoperative complications were balanced between groups with three cases in the TCL as well as the systematic LNE cohort. Postoperative complications included vaginal cuff complications (*n* = 3) as well as postoperative hemorrhage, bowel perforation and excessive drainage of serous fluid from the surgical drainage (*n* = 1, respectively). Table 2 gives an overview of the complications.

**Table 2:** Intra- and postoperative complications

		Total	Syst. LNE	TCL
Intraoperative	No	30 (93.8)	13 (86.7)	17 (100)
Complications	Yes	2 (6.3)	2 (13.3)	0
Postoperative	No	26 (81.3)	12 (80)	14 (82.4
Complications	Yes	6 (18.8)	3 (20)	3 (17.6)
Clavien–Dindo	0	26 (81.3)	12 (80)	14 (82.4)
Grade	1	1 (3.1)	1 (6.7)	0
	2	1 (3.1)	0	1 (5.9)
	3a	1 (3.1)	1 (6.7)	0
	3b	3 (9.4)	1 (6.7)	2 (11.8)

FIGO stage was I in 68.8% of patients (22/32). Five patients (15.6%) had FIGO stage II and III disease, respectively. Positive lymph nodes were found in four patients (12.5%), all of which received systematic pelvic and periaortic LNE.

The mean interval between hysterectomy and secondary PMMR was 34.4 days (6–78; 17.6).

Mean follow-up was 31.7 months (0–92; 28.8) for the whole cohort. In the group of patients who received systematic LNE, the mean observation time was 45.8 months (0–92; 35.4), while it was 19.2 months (0–40; 12.6) in the TCL group.

During the follow-up period, four patients (12.5%) experienced disease recurrence. None of the recurrences can be described as isolated locoregional recurrence. A woman with FIGO II, G3 nodal negative disease developed pulmonary and bone metastases two years after surgery. Postoperatively, she had received chemotherapy and HDR-brachytherapy of the vaginal cuff. She lived for five more years before she died in 2018. Another patient, suffering from FIGO IIIC1, G3 EC was diagnosed with pulmonary metastases as well as pelvic recurrence 3.5 years after surgery despite having received adjuvant pelvic irradiation (50 Gy) and chemotherapy and died two years later. The third recurrence occurred in a woman with FIGO IIIC2, G2 cancer (10/52 nodes, disseminated pelvic and periaortic). She developed inguinal and periaortic lymph node metastases 2.5 years after surgery and died 1.5 years later. This patient had received adjuvant chemotherapy but no irradiation. The last recurrence occurred in a patient with FIGO IIIC2, G2 disease who had received adjuvant irradiation (external + afterloading) as well as chemotherapy but who developed lymphatic and bone metastases 9 months after surgery. She was treated with immunotherapy but lived for only three more months. In total, three out of the four women with positive nodes experienced a recurrence.

In total, five women died during the observation period. In addition to the four mentioned recurring patients, this included a morbidly obese patient (BMI 41.4 kg/m^2^) who experienced an abdominal wall infection with pelvic abscess formation three months after surgery and died despite surgical and intensive care therapy due to septic shock.

To evaluate the effect the concept of cancer field surgery had on adjuvant therapy, we compared the guideline recommendations regarding postoperative radiotherapy with the treatment patients received. As only intermediate/high-risk patients were eligible for inclusion in the analysis, all patients had a guideline recommendation of adjuvant radiotherapy. However, only nine patients (28.1%) received radiotherapy in our collective, thus sparing the morbidity of irradiation for the remaining 71.9% of women. Disease and treatment parameters are summarized in Table 3.

**Table 3:** Disease and treatment parameters

		Total	Syst. LNE	TCL
FIGO stage	FIGO I	22 (68.8)	8 (53.3)	14 (82.4)
	IA	3 (9.4)	1 (6.7)	2 (11.8)
	IB	19 (59.4)	7 (46.7)	12 (70.6)
	FIGO II	4 (12.5)	3 (20)	2 (11.8)
	FIGO III	5 (15.6)	4 (26.7)	1 (5.9)
	IIIA	2 (6.3)	1 (6.7)	1 (5.9)
	IIIC1	1 (3.1)	1 (6.7)	0
	IIIC2	3 (9.4)	3 (20)	0
pT stage	1a	3 (9.4)	1 (6.7)	2 (11.8)
	1b	20 (62.5)	8 (53.3)	12 (70.6)
	2	6 (18.8)	4 (26.7)	2 (11.8)
	3a	2 (6.3)	1 (6.7)	1 (5.9)
	3b	1 (3.1)	1 (6.7)	0
Nodal status	pN0	28 (87.5)	11 (73.3)	17 (100)
	pN1	1 (3.1)	1 (6.7)	0
	pN2	2 (9.4)	3 (20)	0
Histopathologic grading	G1	4 (12.5)	1 (6.7)	3 (17.6)
	G2	20 (62.5)	9 (60)	11 (64.7)
	G3	8 (25)	5 (33.3)	3 (17.6)
Adjuvant radiotherapy	No	23 (71.9)	7 (46.7)	16 (93.7)
	Yes	9 (28.1)	8 (53.3)	1 (6.3)
Adjuvant chemotherapy	No	20 (62.5)	8 (53.3)	12 (70.6)
	Yes	12 (37.5)	7 (46.7)	5 (29.4)
Distant metastases	No	28 (87.5)	11 (73.3)	17 (100)
	Yes	4 (12.5)	4 (26.7)	0
Isolated pelvic recurrence	No	32 (100)	15 (100)	17 (100)
	Yes	0	0	0
Death	No	27 (84.4)	10 (66.7)	17 (100)
	Yes	5 (15.6)	5 (33.3)	0

## Discussion

We present here the first clinical data on the concept of secondary PMMR and lymphadenectomy in endometrial cancer following prior hysterectomy.

Our data indicate the general feasibility of the procedure with an intraoperative complication rate of 6.3%. This rate is substantially higher than those we reported for benign robotic hysterectomy as well as primary PMMR + TCL [6, 28], perhaps reflecting the increased complexity of the procedure following prior hysterectomy. Of note, both intraoperative complications occurred during systematic pelvic and periaortic lymphadenectomy, once more confirming the known surgical morbidity of this procedure. TCL, however, could be performed without intraoperative complications in all patients.

Also, the postoperative complication rate is higher than one would expect in primary surgery for endometrial cancer. In our 2021 publication of 51 EC patients treated with PMMR + TCL, we observed a postoperative complication rate of 13.7%. Again, it can be expected that the status post hysterectomy increases surgical morbidity in comparison to primary operated patients. Especially vaginal cuff insufficiencies might be higher when the vaginal cuff is opened and re-sutured after prior hysterectomy. However, the rate of 12.5% complications of Clavien–Dindo Grade 3 and higher seems reasonable when compared to data from the literature. In a randomized controlled trial of laparoscopic vs. robotically assisted surgery in EC including 99 patients, Mäenpää et al. found a rate of 10% major early complications in the robotic group [29]. In a retrospective cohort study of 1433 women with a diagnosis of complex atypical hyperplasia and endometrial cancer managed by minimally invasive hysterectomy and surgical staging from January 2009 to January 2014, Barrie et al. reported a postoperative complication rate (any grade) of 21.7% in the robotic cohort[30].

The overall recurrence rate in our cohort was 12.5% during a mean observation time of 31.7 months. This is in line with the general recurrence rate of around 13% for endometrial cancer of all risk stratifications [31]. As most recurrences occur during the first two years after primary treatment, we do not expect a significant increase in recurrences in a longer observational period [32, 33]. It must be noted that even though postoperative radiotherapy was spared in 72% of patients, no isolated locoregional recurrence occurred, indicating the achievement of excellent locoregional tumor control by cancer field surgery in these intermediate- and high-risk tumors. Seventy-five percent (3/4) of the patients who recurred did so despite having received postoperative irradiation. The patient who did not receive radiotherapy but developed a recurrence was an extremely high-risk case (FIGO IIIC, 10 lymph nodes involved) and recurred with inguinal lymph node metastases which represent distant metastases and could not be avoided by irradiation.

In conclusion, our findings suggest that cancer field surgery via secondary PMMR and LNE in intermediate/high-risk EC following simple hysterectomy is feasible. In addition, it seems to provide equally excellent locoregional control without adjuvant radiotherapy even when performed as a secondary procedure following prior hysterectomy.

However, postoperative morbidity is relevantly increased compared to primary surgery due to the complexity of the procedure and the specific challenges of the recently operated situs. Moreover, this study represents a small number of cases spanning a long time period. As the approach to lymphadenectomy has changed between 2011 and 2021, the collective is not homogenous as to the type of LNE performed. These results can, therefore, only serve as hypothesis generating. Thus, our primary strategy is to offer PMMR + TCL to all EC patients regardless of preoperative risk assessment to ensure the benefits of cancer field surgery without increasing morbidity.

## Data Availability

The originial data are available from the authors on request.

## References

[1] Ferlay J, Steliarova-Foucher E, Lortet-Tieulent J, Rosso S, Coebergh JW, Comber H (2013). Cancer incidence and mortality patterns in Europe: estimates for 40 countries in 2012. Eur J Cancer.

[2] Robert Koch-InstitutGdeKiDeVH (2021). Krebs in Deutschland für 2017/2018.

[3] Lim MC, Won YJ, Ko MJ, Kim M, Shim SH, Suh DH (2019). Incidence of cervical, endometrial, and ovarian cancer in Korea during 1999–2015. J Gynecol Oncol.

[4] Leitlinienprogramm Onkologie (Deutsche Krebsgesellschaft DK, AWMF). Diagnostik, Therapie und Nachsorge der Patientinnen mit Endometriumkarzi- nom, Kurzversion 1.0, 2018, AWMF Registernummer: 032/034-OL. 2018. http://www.leitlinienprogramm-onkologie.de/leitlinien/endometriumkarzinom/. Accessed 19 Nov 2018

[5] Leitlinienprogramm Onkologie (Deutsche Krebsgesellschaft DK, AWMF). S3-Leitlinie Endometriumkarzinom, Langversion 2.0, 2022, AWMF- Registernummer: 032/034-OL. c2022. https://www.leitlinienprogramm-onkologie.de/leitlinien/endometriumkarzinom/. Accessed 14 Jun 2013

[6] Buderath P, Rusch P, Mach P, Kimmig R (2021). Cancer field surgery in endometrial cancer: peritoneal mesometrial resection and targeted compartmental lymphadenectomy for locoregional control. J Gynecol Oncol.

[7] Kimmig R, Buderath P, Rusch P, Aktas B (2017). Technique of ICG-guided targeted compartmental pelvic lymphadenectomy (TCL) combined with Pelvic Peritoneal Mesometrial resection (PMMR) for locoregional control of endometrial cancer—a proposal. Gynecol Oncol Rep.

[8] Kimmig R, Iannaccone A, Aktas B, Buderath P, Heubner M (2016). Embryologically based radical hysterectomy as peritoneal mesometrial resection (PMMR) with pelvic and para-aortic lymphadenectomy for loco-regional tumor control in endometrial cancer: first evidence for efficacy. Arch Gynecol Obstet.

[9] Kimmig R, Aktas B, Buderath P, Wimberger P, Iannaccone A, Heubner M (2013). Definition of compartment-based radical surgery in uterine cancer: modified radical hysterectomy in intermediate/high-risk endometrial cancer using peritoneal Mesometrial resection (PMMR) by M Höckel translated to robotic surgery. World J Surg Oncol.

[10] Wolpert L (1969). Positional information and the spatial pattern of cellular differentiation. J Theor Biol.

[11] Dahmann C, Oates AC, Brand M (2011). Boundary formation and maintenance in tissue development. Nat Rev Genet.

[12] Höckel M (2015). Morphogenetic fields of embryonic development in locoregional cancer spread. Lancet Oncol.

[13] Höckel M, Hentschel B, Horn LC (2014). Association between developmental steps in the organogenesis of the uterine cervix and locoregional progression of cervical cancer: a prospective clinicopathological analysis. Lancet Oncol.

[14] Höckel M, Trott S, Dornhofer N, Horn LC, Hentschel B, Wolf B (2018). Vulvar field resection based on ontogenetic cancer field theory for surgical treatment of vulvar carcinoma: a single-centre, single-group, prospective trial. Lancet Oncol.

[15] Jeong SY, Park JW, Nam BH, Kim S, Kang SB, Lim SB (2014). Open versus laparoscopic surgery for mid-rectal or low-rectal cancer after neoadjuvant chemoradiotherapy (COREAN trial): survival outcomes of an open-label, non-inferiority, randomised controlled trial. Lancet Oncol.

[16] Yang Y, Oliver G (2014). Development of the mammalian lymphatic vasculature. J Clin Invest.

[17] Höckel M (2001). Total mesometrial resection: nerve-sparing extended radical abdominal hysterectomy. Zentralbl Gynakol.

[18] Höckel M, Horn LC, Tetsch E, Einenkel J (2012). Pattern analysis of regional spread and therapeutic lymph node dissection in cervical cancer based on ontogenetic anatomy. Gynecol Oncol.

[19] Höckel M, Wolf B, Schmidt K, Mende M, Aktas B, Kimmig R (2019). Surgical resection based on ontogenetic cancer field theory for cervical cancer: mature results from a single-centre, prospective, observational, cohort study. Lancet Oncol.

[20] Buderath P, Stukan M, Ruhwedel W, Strutas D, Feisel-Schwickardi G, Wimberger P (2022). Total mesometrial resection (TMMR) for cervical cancer FIGO IB-IIA: first results from the multicentric TMMR register study. J Gynecol Oncol.

[21] Frost JA, Webster KE, Bryant A, Morrison J (2017). Lymphadenectomy for the management of endometrial cancer. Cochrane Database Syst Rev.

[22] Rossi EC, Kowalski LD, Scalici J, Cantrell L, Schuler K, Hanna RK (2017). A comparison of sentinel lymph node biopsy to lymphadenectomy for endometrial cancer staging (FIRES trial): a multicentre, prospective, cohort study. Lancet Oncol.

[23] Abu-Rustum NR (2014). Update on sentinel node mapping in uterine cancer: 10-year experience at Memorial Sloan-Kettering cancer center. J Obstet Gynaecol Res.

[24] Papadia A, Imboden S, Siegenthaler F, Gasparri ML, Mohr S, Lanz S (2016). Laparoscopic indocyanine green sentinel lymph node mapping in endometrial cancer. Ann Surg Oncol.

[25] How JA, O'Farrell P, Amajoud Z, Lau S, Salvador S, How E (2018). Sentinel lymph node mapping in endometrial cancer: a systematic review and meta-analysis. Minerva Ginecol.

[26] Frumovitz M, Plante M, Lee PS, Sandadi S, Lilja JF, Escobar PF (2018). Near-infrared fluorescence for detection of sentinel lymph nodes in women with cervical and uterine cancers (FILM): a randomised, phase 3, multicentre, non-inferiority trial. Lancet Oncol.

[27] Dindo D, Demartines N, Clavien PA (2004). Classification of surgical complications: a new proposal with evaluation in a cohort of 6336 patients and results of a survey. Ann Surg.

[28] Buderath P, Kimmig R, Dominowski L, Mach P (2022). Hysterectomy in benign conditions: a 20-year single-center retrospective on the development of surgical techniques. Arch Gynecol Obstet.

[29] Maenpaa MM, Nieminen K, Tomas EI, Laurila M, Luukkaala TH, Maenpaa JU (2016). Robotic-assisted vs traditional laparoscopic surgery for endometrial cancer: a randomized controlled trial. Am J Obstet Gynecol.

[30] Barrie A, Freeman AH, Lyon L, Garcia C, Conell C, Abbott LH (2016). Classification of postoperative complications in robotic-assisted compared with laparoscopic hysterectomy for endometrial cancer. J Minim Invasive Gynecol.

[31] Fung-Kee-Fung M, Dodge J, Elit L, Lukka H, Chambers A, Oliver T (2006). Follow-up after primary therapy for endometrial cancer: a systematic review. Gynecol Oncol.

[32] Siegenthaler F, Lindemann K, Epstein E, Rau TT, Nastic D, Ghaderi M (2022). Time to first recurrence, pattern of recurrence, and survival after recurrence in endometrial cancer according to the molecular classification. Gynecol Oncol.

[33] Bendifallah S, Ouldamer L, Lavoue V, Canlorbe G, Raimond E, Coutant C (2017). Patterns of recurrence and outcomes in surgically treated women with endometrial cancer according to ESMO-ESGO-ESTRO consensus conference risk groups: results from the FRANCOGYN study Group. Gynecol Oncol.

